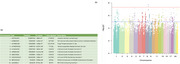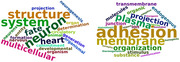# Multivariate non‐parametric GWAS identify novel variants linked to regional WMH via adhesion, membrane organization, and neurodevelopmental pathways

**DOI:** 10.1002/alz70862_110148

**Published:** 2025-12-23

**Authors:** Kahina Baouche, Patricia Genius, Blanca Rodríguez‐Fernández, Tavia E Evans, Jordi Huguet, Carolina Minguillón, Manel Esteller, Arcadi Navarro, Juan Domingo Gispert, Diego Garrido‐Martín, Natalia Vilor‐Tejedor

**Affiliations:** ^1^ Barcelonaβeta Brain Research Center (BBRC), Pasqual Maragall Foundation, Barcelona Spain; ^2^ Centre for Genomic Regulation (CRG), The Barcelona Institute for Science and Technology, Barcelona Spain; ^3^ École Centrale de Nantes, Nantes France; ^4^ Centre for Genomic Regulation (CRG), Barcelona Institute of Science and Technology (BIST), Barcelona Spain; ^5^ IMIM (Hospital del Mar Medical Research Institute), Barcelona Spain; ^6^ Hospital del Mar Research Institute, Barcelona Spain; ^7^ Radboud University Medical Center, Nijmegen Netherlands; ^8^ BarcelonaBeta Brain Research Center (BBRC), Barcelona Spain; ^9^ Barcelonaβeta Brain Research Center, Barcelona, Spain, Barcelona Spain; ^10^ Institució Catalana de Recerca i Estudis Avançats (ICREA), Barcelona Spain; ^11^ Josep Carreras Leukaemia Research Institute (IJC), Badalona, Barcelona Spain; ^12^ Physiological Sciences Department, School of Medicine and Health Sciences, University of Barcelona (UB), Barcelona, Catalonia Spain; ^13^ Institute of Evolutionary Biology (CSIC‐UPF) Universitat Pompeu Fabra, Barcelona Spain; ^14^ Center for Genomic Regulation, Barcelona Spain; ^15^ Department of Genetics, Microbiology and Statistics, Universitat de Barcelona (UB), Barcelona Spain; ^16^ Barcelonaβeta Brain Research Center (BBRC), Barcelona Spain

## Abstract

**Background:**

White Matter Hyperintensities (WMH) are key radiological markers associated with cognitive decline and an increased risk of Alzheimer’s disease (AD). Traditional genome‐wide association studies (GWAS) often overlook subtle genetic contributors to WMH burden due to their reliance on normal distribution assumptions. Moreover, employing multivariate models enhances detection capabilities, particularly when investigating phenotypes with a shared genetic basis. This study aims to employ a non‐parametric, multivariate GWAS approach to delineate the genetic factors influencing regional WMH volumes, advancing our understanding of their role in neurodegeneration.

**Method:**

We analyzed data from 1,388 cognitively unimpaired (CU) participants in the ALFA study (mean age 55.71 (6.71), 61.31% women). Regional WMH volumes, classified based on ventricular proximity into periventricular, deep, and juxtacortical categories, were quantified using T1‐weighted and T2‐FLAIR MRI scans through an automatic Bayesian algorithm. Genetic data were obtained using the Neurochip array (Illumina platform) and imputed using the HRC version r1.1 panel within the Michigan imputation server. We conducted a multivariate asymptotic non‐parametric test of association (MANTA), with regional WMH volumes as the outcomes. The models were adjusted for age, sex, years of education, total intracranial volume, and the first five genetic principal components. The top 1000 genetic variants underwent functional annotation and gene‐set enrichment analyses to identify novel biological pathways impacting WMH. Comparative analysis was performed using parametric multivariate analysis of variance (MANOVA) after a quantile normalisation transformation of regional WMH volumes.

**Result:**

MANTA identified 5 loci surpassing the genome‐wide significance threshold (nominal pvalue<1·10‐8)[Figure 1]. Novel genetic variants were identified compared to classic GWAS, indicating that non‐parametric multivariate assessment may have higher sensitivity in detecting associations across multiple chromosomes. Enrichment analysis featured terms related to cellular adhesion, membrane organization and neurodevelopmental pathways[Figure 2], indicating that these biological processes are significantly involved in regional WMH variations.

**Conclusion:**

Non‐parametric multivariate GWAS effectively identified region‐specific genetic vulnerabilities, surpassing traditional methods in detecting significant loci. This approach elucidates critical biological pathways, leading to cerebrovascular burden in at‐risk CU individuals, demonstrating the value of advanced statistical models for analyzing critical non‐normally distributed endophenotypes related to cognition and AD.